# Supplementary material for: Rapid and unconditional parametric reset protocol for tunable superconducting qubits
Source: Nat Commun. 2021 Oct 11;12:5924. doi: 10.1038/s41467-021-26205-y (PMC8505451; doi:10.1038/s41467-021-26205-y)
Supplement: Supplementary file 1 — Supplementary Information [file 41467_2021_26205_MOESM1_ESM.pdf]

Supplementary material for Rapid and Unconditional Parametric Reset for Tunable Superconducting Qubits

Yu Zhou,[1,*] Zhenxing Zhang,[1,*] Zelong Yin,[1] Sainan Huai,[1] Xiu Gu,[1] Xiong Xu,[1] Jonathan Allcock,[1] Fuming Liu,[1] Guanglei Xi,[1] Qiaonian Yu,[1] Hualiang Zhang,[1] Mengyu Zhang,[1] Hekang Li,[2,3] Xiaohui Song,[2,3] Zhan Wang,[2,3] Dongning Zheng,[2,3] Shuoming An,[1,†] Yarui Zheng,[1] and Shengyu Zhang[1]

[1] *Tencent Quantum Laboratory, Tencent, Shenzhen, Guangdong 518057, China*

[2] *Beijing National Laboratory for Condensed Matter Physics, Institute of Physics, Chinese Academy of Sciences, Beijing 100190, China*

[3] *School of Physical Sciences, University of Chinese Academy of Sciences, Beijing 100049, China*

SUPPLEMENTARY NOTE 1. DEVICE PARAMETERS

	Q1	Q2	Q3
$\omega_r/2\pi$ (GHz)	6.441	6.553	6.686
$\omega_q^{\mathrm{max}}/2\pi$ (GHz)	5.784	5.393	5.813
$\omega_q^{\mathrm{idle}}/2\pi$ (GHz)	5.783 (5.780*)	5.005((4.999*)	5.686 (5.811*)
T_1 (μs)	11.5 (5.34*)	11.4 (5.66*)	9.04 (10.1*)
T_2^* (μs)	11.0 (13.6*)	1.05 (1.03*)	0.89 (25.14*)
$1/\kappa_r$ (ns)	50	34	33
$g_{qr}/2\pi$ (MHz)	78	83	89
$J/2\pi$ (MHz)	Q1,Q2:17.5 Q2,Q3:17.6		

Supplementary Table 1. Device parameters. ω_r, ω_q^{max} and ω_q^{idle} denote the frequency of the readout resonator, qubit frequency at the sweet spot, and operation frequency of the qubit, respectively. g_{qr} is the coupling between the qubit and the resonator. J is the coupling strength between neighbouring qubits. * denotes the qubits' parameters in Supplementary Figure 6 b-e and Supplementary Figure 10.

Our experiments are implemented on three transmon qubits – Q1, Q2 and Q3 – with parameters listed in Supplementary Table 1. The resonator frequency $\omega_r/2\pi$ ranges from 6.441 to 6.686 GHz. The maximum frequency $\omega_q/2\pi$ of Q1 (5.784 GHz) is close to Q3 (5.813 GHz), and about 400 MHz higher than Q2 (5.393 GHz). Each resonator's κ_r is measured individually via AC stark shift spectroscopy. Direct adjacent capacitive coupling is around 17.5 MHz.

SUPPLEMENTARY NOTE 2: EXPERIMENTAL SETUP

A schematic of our experimental setup is displayed in Supplementary Fig. 1. The parametric modulation signal is generated by a home-made Arbitrary Waveform Generator (AWG) with sampling rate 2 Gs/s. After 30 dB attenuation, the signal is combined with a DC signal using a home-made bias tee (flux line). For XY and read-in lines, the baseband signal is generated by AWG, and then up-converted to the driving frequency by IQ mixer with a carrier LO signal generated by the microwave source. The read-out signal from the

qubit is first amplified by an impedance-transformed Josephson parametric amplifier(IMPA) [1] and High Electron Mobility Transistors (HEMTs) at 4K. It is further amplified by room-temperature amplifiers and digitized by an analog-to-digital converter (ADC), before being demodulated and analyzed by DAQ FPGA.

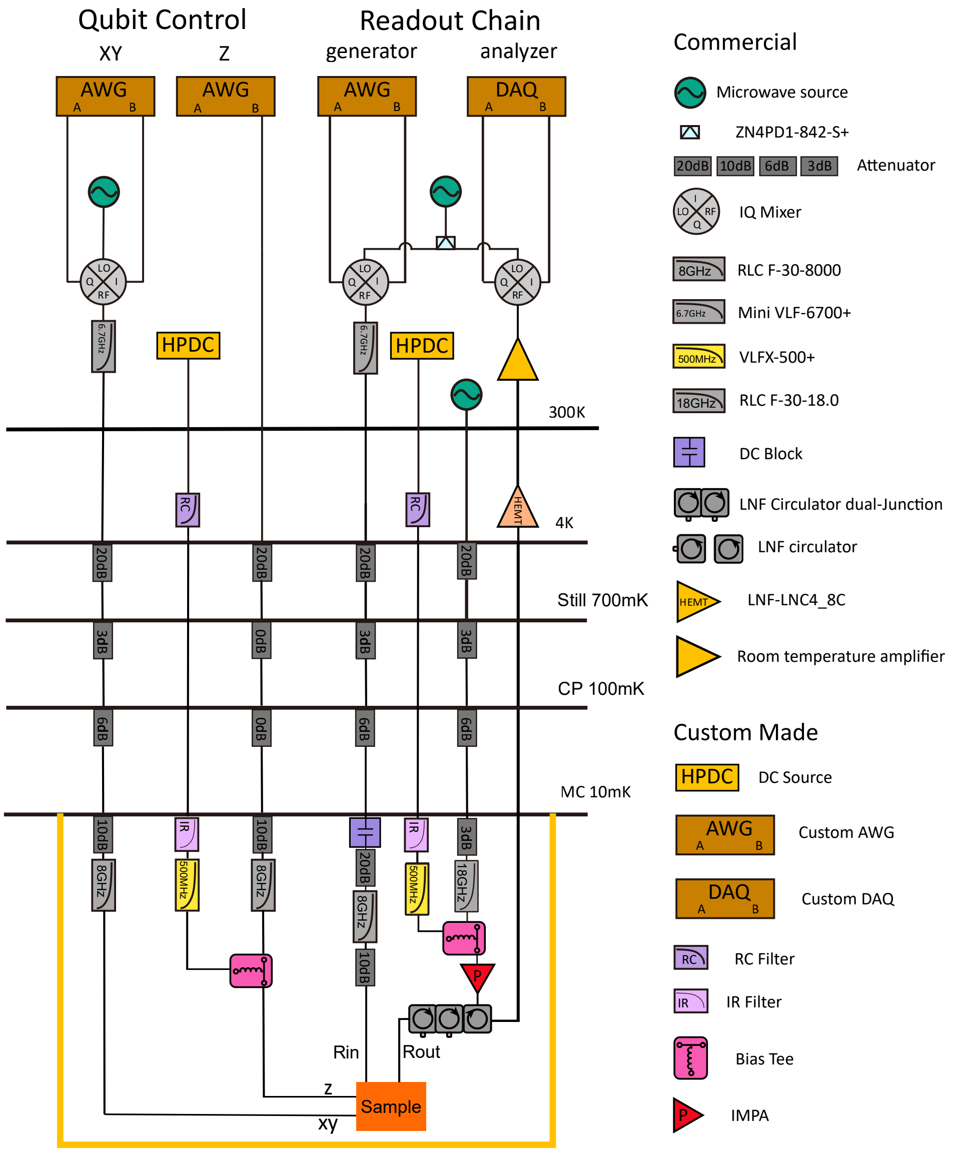

Supplementary Figure 1. Schematic diagram of control electronics and wiring.

SUPPLEMENTARY NOTE 3: PERFORMANCE COMPARISON OF DIFFERENT RESET PROTOCOLS

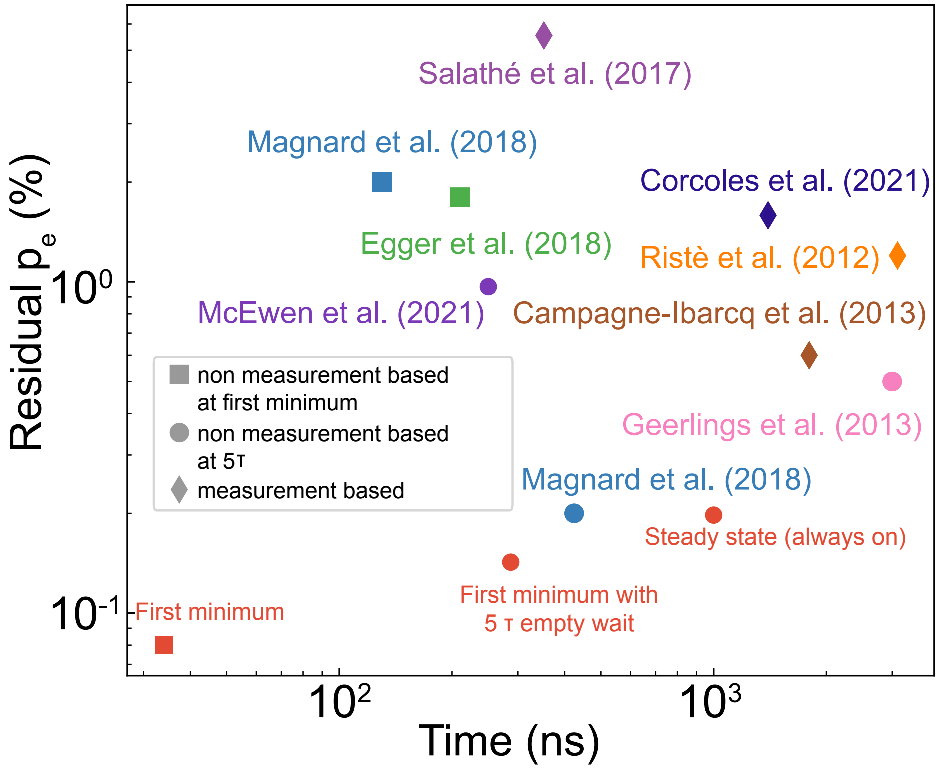

Supplementary Figure 2. Performance comparison of different measurement-based [2–5] and non-measurement-based [6–9] reset protocols. Measurement-based protocols are represented by rhombus. Non-measurement based protocols are represented by squares (first minimum) or circle ($5\tau = 5/\kappa_r$ after the first minimum. The main difference between our two protocols denoted by red circles is whether or not the parametric drive is always on during the reset time.

Supplementary Fig. 2 compares various reset protocols for superconducting qubits in terms of two critical parameters: time (speed) and residual $|e\rangle$ state population (fidelity). Two main types of schemes are included: measurement and non measurement-based protocols. In non measurement-based protocols, we summarize the performance at both the first minimum point, and at the point an additional extra $5\tau = 5/\kappa_r$ after the first minimum (to allow the resonator time to decay to the ground state). In the measurement-based protocols, the time reported is the total duration of the first measurement pulse, the conditional π pulse, and the time interval between the two. The performance and use-case suitability

Protocol type	Performance			Use case suitability		
	Time (ns)	Fidelity	State(s) depleted	Speed requirements	$\lvert f\rangle$ Leakage	Special requirements
One-tone first minimum	34	99.92%	$\lvert e\rangle$	priority	Negligible	No immediate subsequent gate [11,12]
One-tone first minimum+wait $5/\kappa$	284	99.86%	$\lvert e\rangle$	priority	Negligible	Immediate subsequent gate applied(as in Error Correction) [13,14]
One-tone steady (Modulation always on)	1000	99.9%	$\lvert e\rangle$	None	Negligible	None
Two-tone steady (Modulation always on)	600	99.23%	$\lvert e\rangle$	None	Negligible	None
	1000	99.23%	$\lvert e\rangle$ and $\lvert f\rangle$	None	Not negligible [14,15]	None

Supplementary Table 2. Performance and use-case suitability [11–15] for our protocols.

of all the protocols we proposed is summarized in Supplementary Table 2.

SUPPLEMENTARY NOTE 4: TIME EVOLUTION OF EXCITED STATE POPULATION

The population of the resonator-qubit state $|s\rangle$ during the parametric reset process can be expressed as

$$P_{s|s_0}(t) = |\langle s| \exp(-iH_{\text{eff}}t) |s_0\rangle|^2. \tag{1}$$

where $|s_0\rangle$ is the initial state of the system. When the system is prepared in $|e, 0\rangle$, the population of the qubit excited state p_e can be shown to be

$$p_e = P_{e|e}(t) = \begin{cases} e^{-\frac{\kappa_r t}{2}} \left(\frac{\kappa_r t}{4} + 1\right)^2 & |g_n| = \kappa_r/4 \\ e^{-\frac{\kappa_r t}{2}} \left[\cos(Mt) + \frac{\kappa_r}{4M}\sin(Mt)\right]^2 & |g_n| > \kappa_r/4,\ M = \frac{\sqrt{16|g_n|^2 - \kappa_r^2}}{4} \\ e^{-\frac{\kappa_r t}{2}} \left[\cosh(Mt) + \frac{\kappa_r}{4M}\sinh(Mt)\right]^2 & |g_n| < \kappa_r/4,\ M = \frac{\sqrt{\kappa_r^2 - 16|g_n|^2}}{4} \end{cases} \tag{2}$$

When fitting to experimental data, we use the function

$$f(t) = \lambda p_e + \mu \tag{3}$$

where λ and μ are introduced to account for SPAM errors.

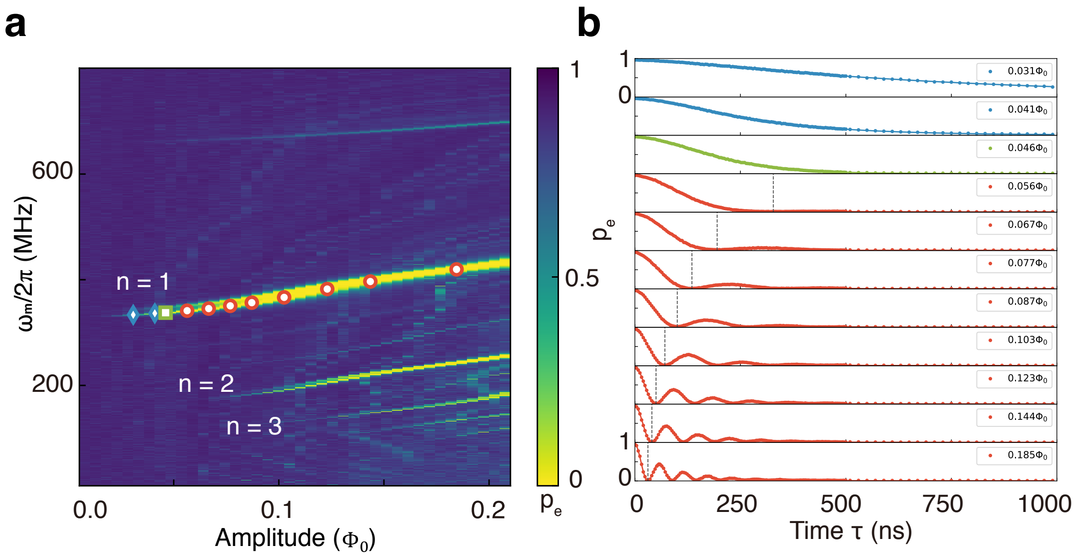

Supplementary Figure 3. Time evolution of different parameters during the parametric reset process. **a** Two dimensional scan map of $|e\rangle$ population, p_e. **b** Population p_e of the excited state $|e\rangle$ as a function of reset duration τ, for the points shown in **a**. Red (blue) symbols represent the underdamped (overdamped) regime, and green symbols correspond to parameters close to the critically damped point. The black dashed lines indicate the first minimum of p_e. Modulation amplitudes are shown in the upper right corner of each panel.

Supplementary Fig. 3a displays the same two-dimensional scan data as Fig. 2b of the main text. At each of the amplitude-frequency parameter pairs corresponding to the colored markers in the $n = 1$ strip, we measure the excited population against p_e as a function of the reset duration τ, with results given in Supplementary Fig. 3b. From top down, the modulation amplitude increases from $0.03\Phi_0$ to $0.185\Phi_0$. Colored dots in the traces are experimental results, and solid lines are fittings to the theoretical model (Supplementary Equation (3)). We use the same λ and μ across the traces, as these parameters are expected to be independent of modulation amplitude.

The traces in blue shows a decay without any observable oscillation. From the fitting, we verify that $|g_n|/4 < \kappa_r$ ($|g_n|/\kappa_r \approx 0.127, 0.195$ respectively), corresponding to the overdamped regime. The population decays faster when the modulation amplitude increases, which agrees with the theoretical prediction (equation (4) in the main text). For the population in green, no oscillation is observed, and the parameter $|g_n|/4\kappa_r$ is approximately 0.268, very close to the crictical value ($|g_n|/4\kappa_r = 0.25$). The red traces are in the underdamped

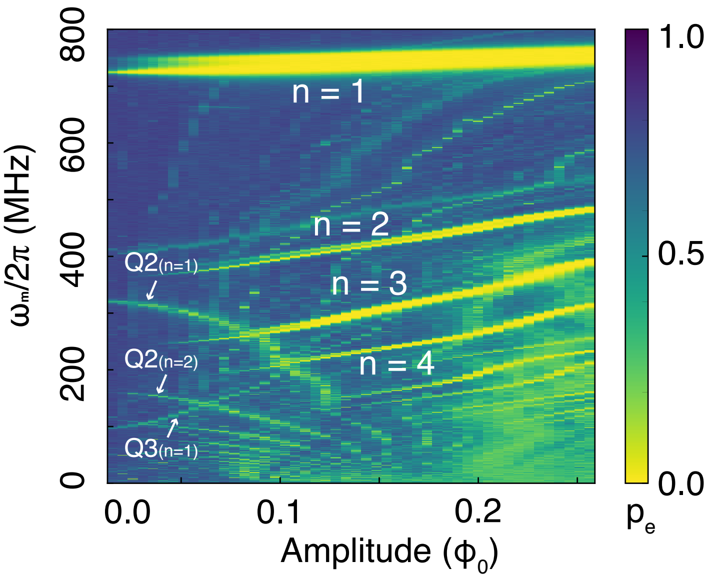

Supplementary Figure 4. Q1 operation point away from sweet spot. Excited state population of Q1 as a function of parametric reset amplitude and frequency.

regime where oscillations can be observed. The dashed black lines in these panels represent the times when the population achieves a minimum for the first time. In the underdamped regime, we see that Rabi oscillations become more pronounced as the modulation amplitude increases, and the time to the first minimum decreases. By fitting to the theoretical model (Supplementary Equation (3)), we find that the parameter $|g_n|/4\kappa_r$ increases from 0.421 to 2.195 as the modulation amplitude increases from $0.056\Phi_0$ to $0.185\Phi_0$.

SUPPLEMENTARY NOTE 5: Q1 OPERATION POINT AWAY FROM THE SWEET SPOT

When the operation point for qubit Q1 is away from the sweet spot, the $|e\rangle$ state population is monitored by varying the modulation frequency ω and amplitude A, as displayed in Supplementary Fig. 4. Several strip-shaped regions labeled $n = 1, 2, 3$ can be seen which correspond to the n-th order modulations, in which parametric resets can be performed. There are also several strip-shaped lines in the bottom left corner, which we attribute to parametric-modulation-induced qubit-qubit interactions with qubits Q2 and Q3. Supporting this assumption is the observation that, with other scan parameters unchanged, if Q2 or Q3 is tuned far from their sweet spots using DC bias, the corresponding strips disappear.

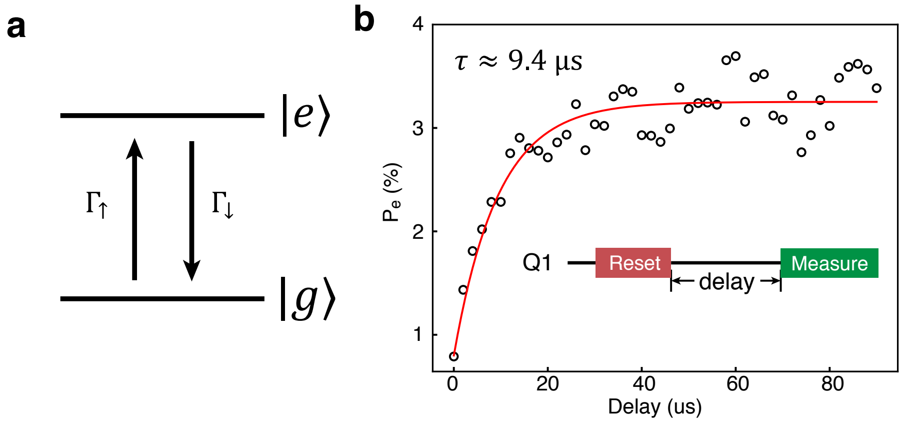

Supplementary Figure 5. a Rate equation model with excited rate $\Gamma_{\uparrow}$ and qubit reset rate $\Gamma_{\downarrow}$. **b** Re-thermalization after reset.

SUPPLEMENTARY NOTE 6: RESIDUAL EXCITED POPULATION LIMIT ESTIMATION AND THE THERMAL OCCUPATION OF RESONATOR

Supplementary Fig. 5a illustrates the rate equation model for a qubit with ground and excited states $|g\rangle$ and $|e\rangle$, and excitation and reset rates $\Gamma_{\uparrow}$ and $\Gamma_{\downarrow}$. When the system reaches thermal equilibrium, the final residual $|e\rangle$ population p_e is given by $p_e = \Gamma_{\uparrow}/(\Gamma_{\uparrow}+\Gamma_{\downarrow})$. Before reset, p_e is measured to be around 2.38% from Rabi population measurement (RPM) as described in the main text. From the measured T_1 we deduce that $1/T_1 = \Gamma_{\uparrow}+\Gamma_{\downarrow} = 86.6\text{kHz}$. When applying parametric reset, $\Gamma_{\uparrow}$ remains the same value while $\Gamma_{\uparrow} + \Gamma_{\downarrow}$ is 10 MHz in the underdamped region ($\kappa_r/2$, the exponential term in Supplementary Equation (2) when $|g_n| > \kappa_r/4$). Accordingly, $p_{e(\text{reset})}$ drops to 0.02%, a rough theoretical estimate of the residual excitation population limit. After the reset, the system will be re-thermalized to the initial steady state as shown in Supplementary Fig. 5b. The raw data is fit to a single exponential function with time constant 9.4 μs.

When the system begins with $|g\rangle$, there is no experimental observation of $|e\rangle$ or $|f\rangle$ population. Based on our state readout fidelity, we conclude that the observed excitation probability below 0.1%. The total number of excitations should be conserved during the reset protocol, and the reset frequency (hundreds of MHz) is much smaller than the qubit and resonator energy gap. The likelihood of re-excitation during the reset modulation should thus be negligible.

When the average thermal population of the resonator $\overline{n} \ll 1$, the thermal population induced dephasing is propotional to $\overline{n}$ [10], i.e.

$$\Gamma_\phi = \frac{1}{T_2^*} = \frac{\overline{n}\kappa\chi^2}{\chi^2 + \kappa^2} \tag{4}$$

where κ is the resonator linewidth and $\chi = g_{qr}^2/(\Delta(1 + \Delta/\eta))$ is the dispersive shift. From the measured value of $T_2^*, g_{qr}, \eta, \kappa, \Delta = \omega_r - \omega_q$ in Supplementary Table 1 and main text, and assuming that thermal population is the only source of dephasing, we deduce $\overline{n} \leq 0.01$ (corresponding to a negligible stark shift of 51.5 kHz). This estimate gives an upper bound on the thermal population.

SUPPLEMENTARY NOTE 7: RAMSEY EXPERIMENTS TO STUDY THE EFFECTS OF THE PARAMETRIC DRIVE ON NEIGHBOURING QUBITS

To investigate the effect of the parametric modulation pulse on neighbouring qubits, we perform a series of Ramsey measurement on one qubit. During the interval τ – ranging from 100 to 1000 ns – between two $\pi/2$-pulses, on another qubit(s) we either apply parametric modulation or, for comparison, do nothing. The pulse sequence is presented in Supplementary Fig. 6a. The amplitude and phase of each Ramsey fringe can be extracted by sinusoidal fitting. We investigate the following cases: Supplementary Fig. 6b,c: Ramsey on Q2 while Q1 and Q3 are reset simultaneously. Supplementary Fig. 6d,e: Reset on Q2 and Ramsey on Q1 or Q3 respectively. Supplementary Fig. 6f,g: Reset on Q1 and Ramsey on Q2 or Q3, respectively. In all cases, we observe that the Ramsey amplitude decreases with τ, which is reasonable due to inevitable dephasing. More importantly, no additional degradation of the coherence is observed when the parametric reset modulation is applied on neighbouring qubits. With τ increasing from 100 ns to 1000 ns, the phase remains steady with fluctuations below 1 rad. From a linear fitting to each case, we determine that the frequency shift due to the parametric modulation pulse is in the tens of kHz. These experiments demonstrate that our parametric reset protocol has negligible effects on neighbouring qubits in terms of both coherence and frequency (crosstalk).

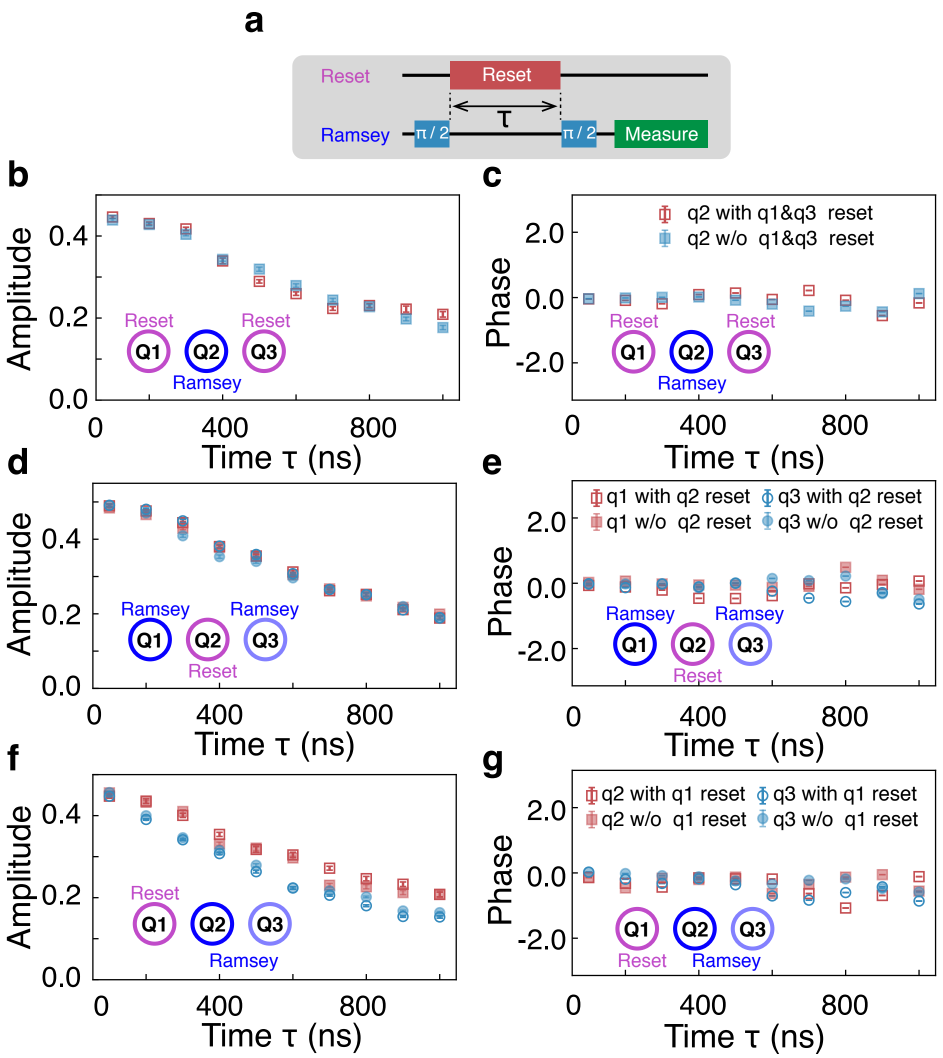

Supplementary Figure 6. Ramsey based experiments. **a** Pulse sequence: Ramsey measurement on one qubit by changing the phase of the second $\pi/2$-pulse. During the interval τ between two $\pi/2$-pulses, the other qubit is reset. The Ramsey amplitude and phase are extracted by fitting sinusoids to the Ramsey fringes at each τ. **b,c** Ramsey on Q2 while Q1 and Q3 are reset simultaneously.**d,e** Reset on Q2 and Ramsey on Q1 or Q3, respectively. **f,g** Reset on Q1 and Ramsey on Q2 or Q3, respectively. All error bars are statistical (± 1 s.d.). No additional effects of decoherence or frequency shifts are observed.

SUPPLEMENTARY NOTE 8: RESET OF THE $|f\rangle$ STATE

Here, we briefly describe the theory behind the two-tone reset protocol used in the main text to reset both the $|e\rangle$ and $|f\rangle$ states. The Hamiltonian of the qubit-resonator system can be expressed as:

$$H_S = \sum_n (n\omega_q(t) + \eta_n) |n\rangle\langle n|_q + \omega_r a^\dagger a + g_{qr} \sum_n \sqrt{n+1}(|n\rangle\langle n+1|_q a^\dagger + |n+1\rangle\langle n|_q a) \quad (5)$$

where $\omega_q(t)$ is the time dependent qubit frequency under modulation, a is the resonator annihilation operator, g_{qr} is the coupling strength between qubit and resonator, and η_n is the anharmonicity of the qubit, $\eta_{0,1} = 0, \eta_2 = \eta$. We consider the three lowest levels of the resonator, and during the modulation of the qubit, the frequency $\omega_q(t)$ can be expanded as

$$\omega_q(t) = \overline{\omega_q} + \sum_{k=1}^{M} A_k \cos \omega_k t \quad (6)$$

In the interaction picture, the Hamiltonian reads:

$$H_{\text{int}}(t) = g_{qr} \exp\left(i \sum_{k=1}^{M} \frac{A_k}{\omega_k} \sin \omega_k t \right) \left[e^{i\overline{\Delta}t} |e0\rangle\langle g1| + e^{i(\overline{\Delta}+\eta)t} |f0\rangle\langle e1| + e^{i\overline{\Delta}t} |e1\rangle\langle g2| + h.c. \right] \quad (7)$$

where $\overline{\Delta} = \overline{\omega_q} - \omega_r$ Expanding the exponential term $\exp\Big(i \sum_{k=1}^{M} \frac{A_k}{\omega_k} \sin(\omega_k t) \Big)$ using the identity $e^{iy\sin x} = \sum_{n\in\mathbb{Z}} J_n(y) e^{inx}$, we obtain:

$$\begin{aligned} \exp\left(i \sum_{k=1}^{M} \frac{A_k}{\omega_k} \sin \omega_k t \right) &= \prod_{k=1}^{M} J_0\left(\frac{A_k}{\omega_k}\right) \\ &+ \sum_{k=1}^{M} J_1\left(\frac{A_k}{\omega_k}\right) (e^{i\omega_k t} - e^{-i\omega_k t}) \left(\prod_{m\neq k} J_0\left(\frac{A_m}{\omega_m}\right) \right) \\ &+ \dots \end{aligned} \quad (8)$$

By ignoring the higher order terms, the Hamiltonian in the interaction picture becomes:

$$H_{\text{int}}(t) \approx \left[g_0 + \sum_{k=1} g_{1,k} (e^{i\omega_k t} - e^{-i\omega_k t}) \right] \left[e^{i\overline{\Delta}t} |e0\rangle\langle g1| + e^{i(\overline{\Delta}+\eta)t} |f0\rangle\langle e1| + e^{i\overline{\Delta}t} |e1\rangle\langle g2| + h.c. \right] \quad (9)$$

where

$$\begin{aligned} g_0 &= g \prod_{k=1}^{M} J_0\left(\frac{A_k}{\omega_k}\right) \\ g_{1,k} &= g J_1\left(\frac{A_k}{\omega_k}\right) \left(\prod_{m\neq k} J_0\left(\frac{A_m}{\omega_m}\right) \right) \end{aligned} \quad (10)$$

From Supplementary Equation (9), when one of the ω_k satisfies $\omega_k = \pm\overline{\Delta}$, a swap between $|e0\rangle$ and $|g1\rangle$ occurs, and the qubit population of $|e\rangle$ can be reset through the resonator. Similarly, when one of the ω_k satifies $\omega_k = \pm(\overline{\Delta} + \eta)$, a swap between $|f0\rangle$ and $|e1\rangle$ is activated, which enables the reset of the $|f\rangle$. As the qubit frequency is modulated with multiple frequencies (Supplementary Equation (6)), the reset of $|e\rangle$ and $|f\rangle$ can be achieved simultaneously through different frequencies.

In the main text, the flux is modulated by two frequencies $\omega_{1,2}$, and the qubit frequency $\omega_q(t)$ has four main Fourier components $2\omega_1, 2\omega_2, \omega_1 \pm \omega_2$. Thus $2\omega_{1,2} = -\Delta$ or $\omega_1 + \omega_2 = -\Delta$ enables the reset of $|e\rangle$, and $2\omega_{1,2} = -\Delta - \eta$ or $\omega_1 + \omega_2 = -\Delta - \eta$ enables the reset of $|f\rangle$. When $\omega_1 = -\Delta/2$ and $\omega_2 = -\Delta/2 - \eta$, both $|e\rangle$ and $|f\rangle$ are reset (rhombus R in Fig.4b in the main text)

The full scan-maps of our two-tone parametric reset are displayed in Supplementary Fig. 7 with 1000 ns reset time and qubit prepared in the $|f\rangle$ state before reset (c.f. Fig. 4b and c in the main text). The first and second rows are experimental and master equation simulation data, respectively, with good agreement between the two. The yellow strips in the P_f scan-map are located in the same positions as the blue strips in the P_e scan-map, which indicates that a swap between $|f0\rangle$ and $|e1\rangle$ – and thus the depletion of $|f\rangle$ – occurs in this region. Similarly, the relative positions of the yellow strips in the P_e map and the blue strips in the P_g map indicate an exchange between $|e0\rangle$ and $|g1\rangle$, corresponding to a reset of the $|e\rangle$ state. Small deviations between experimental and simulated data can be observed in Supplementary Fig. 7, which we attribute to a mismatch between the transfer functions of the z pulse in simulation and experiment.

SUPPLEMENTARY NOTE 9: REPEATED RESET

The data in Supplementary Fig. 8 verifies that our reset protocols can be consistently reapplied without accumulation of errors. In **a**, the qubit is repeatedly prepared in the $|e\rangle$ state and then reset via single-tone modulating pulse. In **b**, the qubit is repeatedly prepared in the $|f\rangle$ and reset via a two-tone modulating pulse. In both figures, the excitation-reset procedure is repeated up to 100 times. No significant errors are observed during these repeated reset processes.

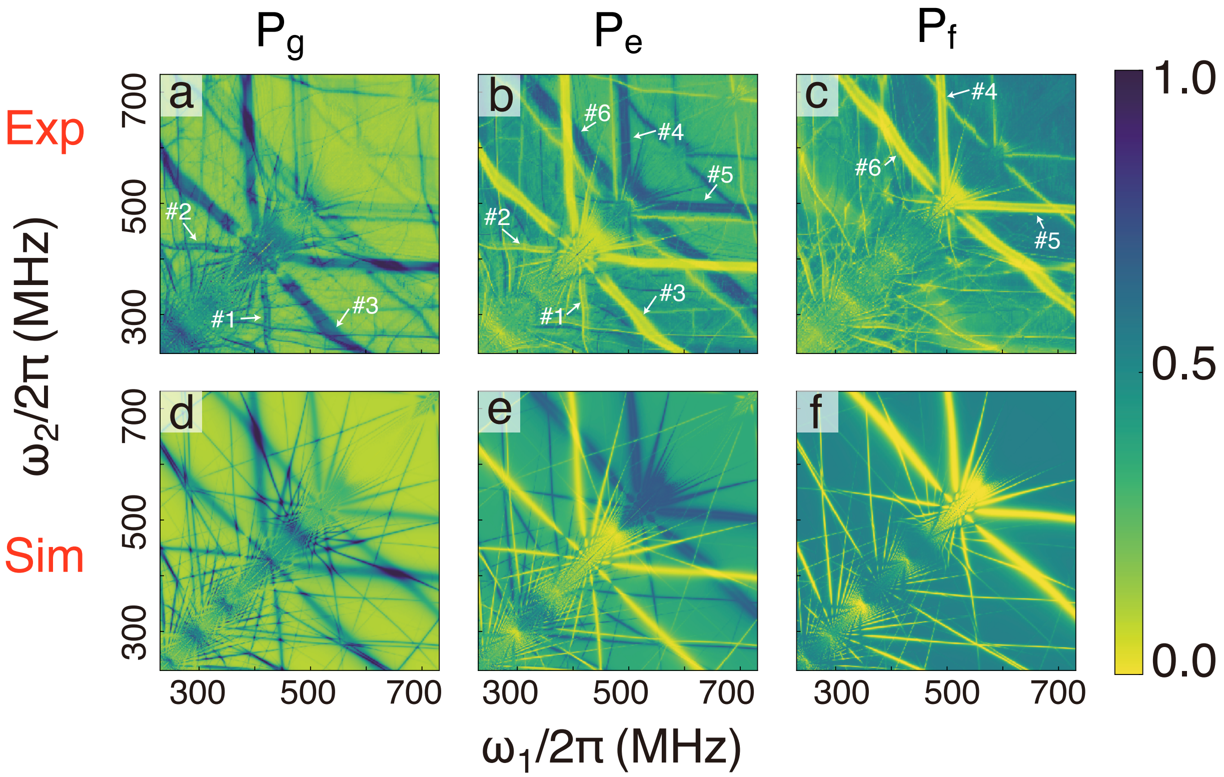

Supplementary Figure 7. two-tone parametric reset **a-c** The qubit is prepared in the $|f\rangle$ state and then reset with 1000 ns two-tone parametric reset pulse, after the reset, the population of P_g,P_e,P_f is measured in **a**, **b**, **c** respectively. **d-f** Master equation simulation of the whole process with the same parameters as in **a-c**

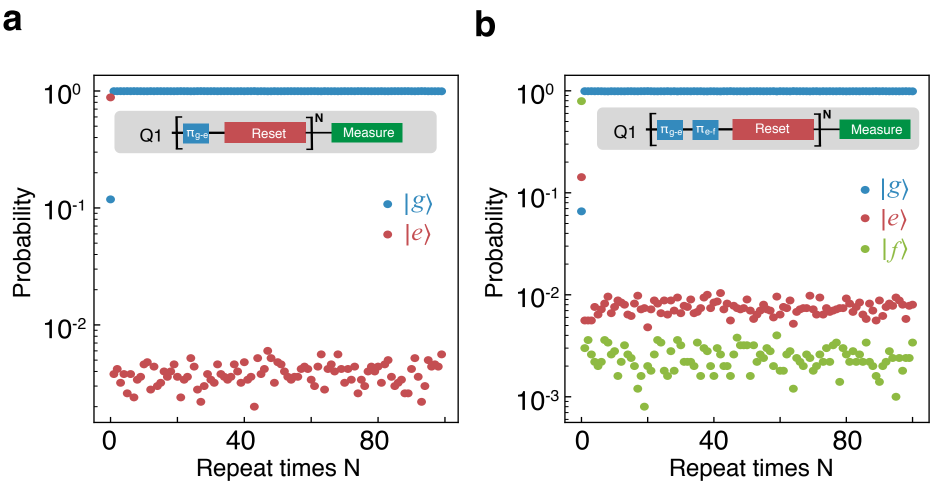

Supplementary Figure 8. Repeated reset. **a** Initial $|e\rangle$ state with single-tone parametric reset repeated for 100 times. **b** Initial $|f\rangle$ state with two-tone parametric reset repeated for 100 times.

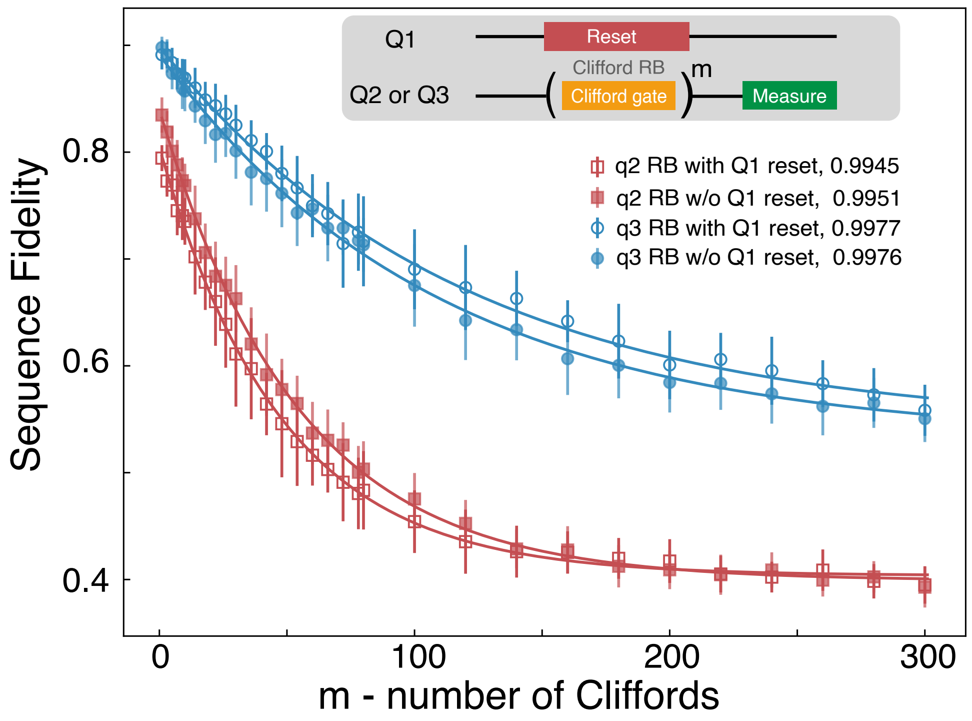

Supplementary Figure 9. Clifford RB on neighbouring qubits. **a** Initial $|e\rangle$ state with single-frequency reset, repeated 100 times. **b** Initial $|f\rangle$ state with two-frequency reset, repeated 100 times. The error bars are statistical (± 1 s.d.) with 30 repetitions.

SUPPLEMENTARY NOTE 10: RB OF THE TWO-TONE PARAMETRIC RESET

The two-tone parametric reset pulse has more frequency components than the one-tone pulse. Those unused frequency components may introduce gate errors in neighbouring qubits. We study these effects in Supplementary Figure 9. During the two-tone reset of Q1, Clifford randomized benchmarking (RB) is performed on the nearest-neighbour (Q2) or next-nearest-neighbour (Q3) qubit. For comparison, we perform RB on Q2 and Q3 without any rest on Q1. The error bars are statistical (± 1 s.d.) with 30 repetitions. We find the average gate fidelity variation between whether the reset is applied or not is 0.06% (0.01%) on Q2 (Q3).

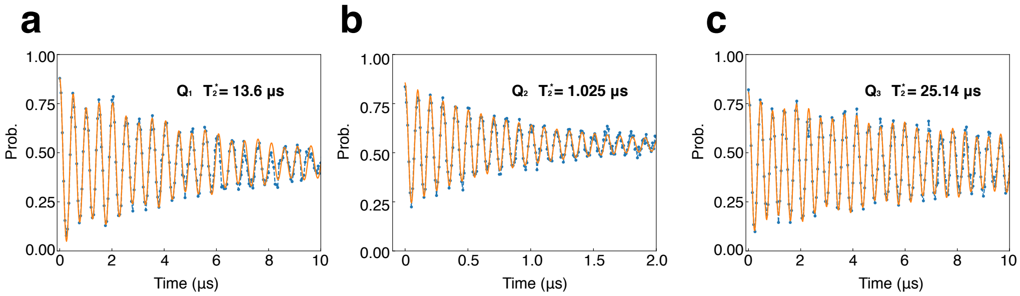

Supplementary Figure 10. Ramsey fringes of the three qubits.

SUPPLEMENTARY NOTE 11: RAMSEY FRINGES FITTING WITH THE BEATING PATTERN

We observed beating patterns in the Ramsey fringes of Q1 and Q3, as shown in Supplementary Figure 10 a and c. We fitted those data by

$$P_t = e^{-t/T_2}(A_1\cos(\omega_1 t + \phi_1) + A_2\cos(\omega_2 t + \phi_1)) + B \tag{11}$$

as in [16]. After removing the T_1 contribution in the fitted T_2, we extracted pure dephasing T_2^* in Supplementary Table 1 (for Ramsey fringes of Q2, no beating pattern is observed, only one oscillation term is used in the fitting). The beating is usually related to the noise in the environment. We performed spin-echo measurements of Q1 and Q3 ,and the beating patterns disappear, suggesting that the noise can be refocused by adding a π pulse between two $\pi/2$ pulses.

* These two authors contributed equally to this work.
† shuomingan@tencent.com
[1] Mutus, J. Y. *et al.* Strong environmental coupling in a josephson parametric amplifier. *Appl. Phys. Lett* **104**, 263513 (2014).

[2] Salathé, Y. *et al.* Low-latency digital signal processing for feedback and feedforward in quantum computing and communication. *Phys. Rev. Appl* **9**, 034011 (2018).

[3] Ristè, D., van Leeuwen, J. G., Ku, H.-S., Lehnert, K. W. & DiCarlo, L. Initialization by measurement of a superconducting quantum bit circuit. *Phys. Rev. Lett* **109**, 050507 (2012).

[4] Campagne-Ibarcq, P. *et al.* Persistent control of a superconducting qubit by stroboscopic measurement feedback. *Phys. Rev. X* **3**, 021008 (2013).

[5] Corcoles, A. D. *et al.* Exploiting dynamic quantum circuits in a quantum algorithm with superconducting qubits. *arXiv preprint arXiv:2102.01682* (2021).

[6] Magnard, P. *et al.* Fast and unconditional all-microwave reset of a superconducting qubit. *Phys. Rev. Lett* **121**, 060502 (2018).

[7] Egger, D. J. *et al.* Pulsed reset protocol for fixed-frequency superconducting qubits. *Phys. Rev. Appl* **10**, 044030 (2018).

[8] Geerlings, K. *et al.* Demonstrating a driven reset protocol for a superconducting qubit. *Phys. Rev. Lett* **110**, 120501 (2013).

[9] McEwen, M. *et al.* Removing leakage-induced correlated errors in superconducting quantum error correction. *Nature communications* **12**, 1–7 (2021).

[10] Clerk, A.A. *et al.* Using a qubit to measure photon-number statistics of a driven thermal oscillator. *Phys. Rev. A* **75**, 042302 (2007).

[11] Kurpiers, P. *et al.* Deterministic quantum state transfer and remote entanglement using microwave photons. *Nature* **558**, 264–267 (2018).

[12] Ma, R. *et al.* A dissipatively stabilized mott insulator of photons. *Nature* **566**, 51–57 (2019).

[13] Fowler, A. G., Mariantoni, M., Martinis, J. M. & Cleland, A. N. Surface codes: Towards practical large-scale quantum computation. *Phys. Rev. A* **86**, 032324 (2012).

[14] Marques, J. F. *et al.* Logical-qubit operations in an error-detecting surface code *arXiv:2102.13071* (2021).

[15] Sank, D. *et al.* Measurement-induced state transitions in a superconducting qubit: Beyond the rotating wave approximation *Phys. Rev. Lett* **117**, 190503 (2016).

[16] Peterer, M.J. *et al.* Coherence and decay of higher energy levels of a superconducting transmon qubit *Phys. Rev. Lett* **114**, 010501 (2015).
